# Inhibition of calcium-activated chloride channel ANO1 suppresses proliferation and induces apoptosis of epithelium originated cancer cells

**DOI:** 10.18632/oncotarget.12524

**Published:** 2016-10-08

**Authors:** Lizhao Guan, Yan Song, Jian Gao, Jianjun Gao, KeWei Wang

**Affiliations:** ^1^ Department of Molecular and Cellular Pharmacology, State Key Laboratory of Natural and Biomimetic Drugs, Peking University School of Pharmaceutical Sciences, Beijing 100191, China; ^2^ Department of Pharmacology, Qingdao University School of Pharmacy, Qingdao 266021, China

**Keywords:** ANO1, proliferation, apoptosis, migration, cancer, CaCC_inh_-A01, T16A_inh_-A01

## Abstract

ANO1, a calcium-activated chloride channel, has been reported to be amplified or overexpressed in tissues of several cancers. However, reports on its roles in tumor progression obtained from cancer cell lines are inconsistent, suggesting that the role of ANO1 in tumorigenesis is likely dependent on either its expression level or cell-type expressing ANO1. To investigate the biological roles of ANO1 in different tumor cells, we, in this study, selected several cancer cell lines and a normal HaCaT cell line with high expression levels of ANO1, and examined the function of ANO1 in these cells using approaches of lentiviral knockdown and pharmacological inhibition. We found that ANO1 knockdown significantly inhibited cell proliferation and induced cell apoptosis in either tumor cell lines or normal HaCaT cell line. Moreover, silencing ANO1 arrested cancer cells at G1 phase of cell cycle. Treatment with ANO1 inhibitor CaCC_inh_-A01 reduced cell viability in a dose-dependent manner. Furthermore, both ANO1 inhibitors CaCC_inh_-A01 and T16A_inh_-A01 significantly suppressed cell migration. Our findings show that ANO1 overexpression promotes cancer cell proliferation and migration; and genetic or pharmacological inhibition of ANO1 induces apoptosis and cell cycle arrest at G1 phase in different types of epithelium-originated cancer cells.

## INTRODUCTION

Calcium-activated chloride channels (CaCCs) are widely expressed in various tissues and involved in important physiological processes such as epithelial secretion, smooth muscle contraction and sensory transduction [1, 2]. ANO1, also known as TMEM16A, was identified as a bona fide CaCC that mediates endogenous calcium-activated chloride current [3–5]. ANO1 is a voltage-sensitive calcium activated chloride channel that is expressed in several epithelial and nonepithelial tissues, playing important physiological roles similar to native CaCCs [6–8]. Besides the physiological significance, ANO1 is also implicated in tumorigenesis since its discovery. *ANO1* gene is located within the 11q13 amplicon, one of the most frequently amplified chromosomal regions in human cancers that is associated with a poor prognosis [9, 10]. Amplification or overexpression of ANO1 has been found in several cancers, including gastrointestinal stromal tumor (GIST), head and neck squamous cell carcinoma (HNSCC), prostate cancer, breast cancer and pancreatic cancer [11–17]. The upregulation of ANO1 has also recently been reported in colon cancer and lung adenocarcinoma [18, 19], and is correlated with poor prognosis of HNSCC and breast cancer [15, 20].

Although ANO1 is considered as a potential tumor biomarker, reports on its roles in tumor progression are inconsistent. It has been shown that ANO1 promotes cell proliferation and tumor growth in HNSCC and breast cancer by activating MAPK signaling pathway and activating EGFR and CAMK signaling respectively [15, 21]. Pro-survival effects have also been shown in some cell lines such as colon cancer cell line SW620 and lung cancer cell line GLC82 [18, 19]. In HNSCC cell lines BHY, HEp-2, SCC-25 and some pancreatic cancer cell lines, ANO1 overexpression or knockdown affects cell migration rather than proliferation [14, 17, 20]. In addition, some studies have also shown that ANO1 has no effect on either cell proliferation or migration [22, 23]. These findings imply that ANO1 effect might be mediated by either same or distinct signaling pathways or cell type-dependent mechanism. Then, the questions arise as to whether different expression levels of ANO1 in different epithelial cells of the same origin differentially affect the cell proliferation and viability, and whether suppressing ANO1 expression and function can have any impact on different epithelium-originated tumor cells.

In the present study, we selected several cell lines with high level of ANO1 expression, and investigated the effect of ANO1 on these cell lines by means of lentiviral knockdown and pharmacological inhibition. We found that silencing ANO1 inhibited cell proliferation and induced apoptosis in all tested cell lines. Treatment with ANO1 inhibitor CaCC_inh_-A01 reduced cell viability whereas inhibitor T16A_inh_-A01 had a little effect on cell viability. Both inhibitors showed inhibitory effect on cell migration. Our findings demonstrate that upregulation of ANO1 promotes cell proliferation and migration; and the pro-survival properties of ANO1 are characterized by different types of epithelial cells, suggesting that effect of ANO1 on epithelial cancer cells is likely mediated by similar signaling pathways.

## RESULTS

### High expression of ANO1 in prostate and colon cancer cell lines

**Figure 1:**
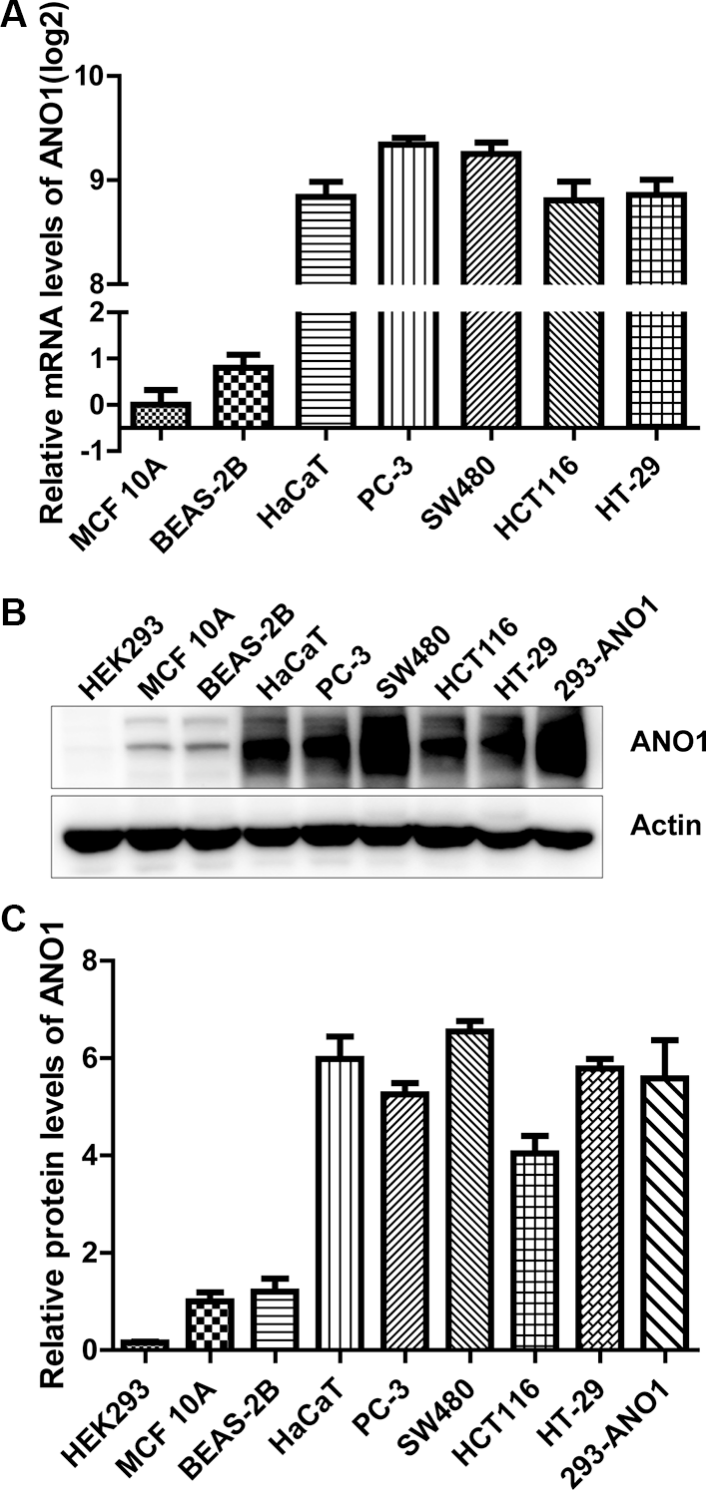
Comparison of ANO1 expression levels in multiple epithelial cell lines (**A**) ANO1 mRNA expression in seven cell lines was determined by quantitative real-time PCR. The mRNA expression of ANO1 was much higher in human keratinocyte HaCaT, prostate cancer cell line PC-3, colon cancer cell lines SW480, HCT116 and HT-29 than that in normal breast epithelium MCF 10A and bronchial epithelial cell line BEAS-2B. (**B**) ANO1 protein expression in the cell lines was detected by Western blot. Higher expression of ANO1 was confirmed in cell lines of HaCaT, PC-3, SW480, HCT116 and HT-29 than MCF 10A and BEAS-2B. HEK293 cells transfected with ANO1 plasmid were used as positive control. (**C**) Quantitative analysis of ANO1 protein expression in cell lines from B.

To investigate the biological function of ANO1, we started detecting the expression levels of ANO1 in several normal and cancer cell lines. The mRNA expression of ANO1 was very low in normal breast epithelial cells MCF 10A and normal bronchial epithelial cells BEAS-2B as examined by real-time PCR. Much higher ANO1 expression was found in human keratinocyte cell line HaCaT, prostate cancer cell line PC-3, and the three colon cancer cell lines SW480, HCT116 and HT-29. ANO1 expression in these cell lines increased more than 2^8^-fold, as compared with MCF 10A cells (Figure 1A). The protein expression of ANO1 was also detected by Western blot (Figure 1B), and quantitative analysis showed about 6-fold elevation in HaCaT and four cancer cell lines, as compared with MCF 10A and BEAS-2B cells (Figure 1C). This result is consistent with the real-time PCR analysis, further confirming the relative high expression of ANO1 in HaCaT and prostate and colon cancer cell lines.

### Knockdown of ANO1 inhibits cell proliferation

**Figure 2:**
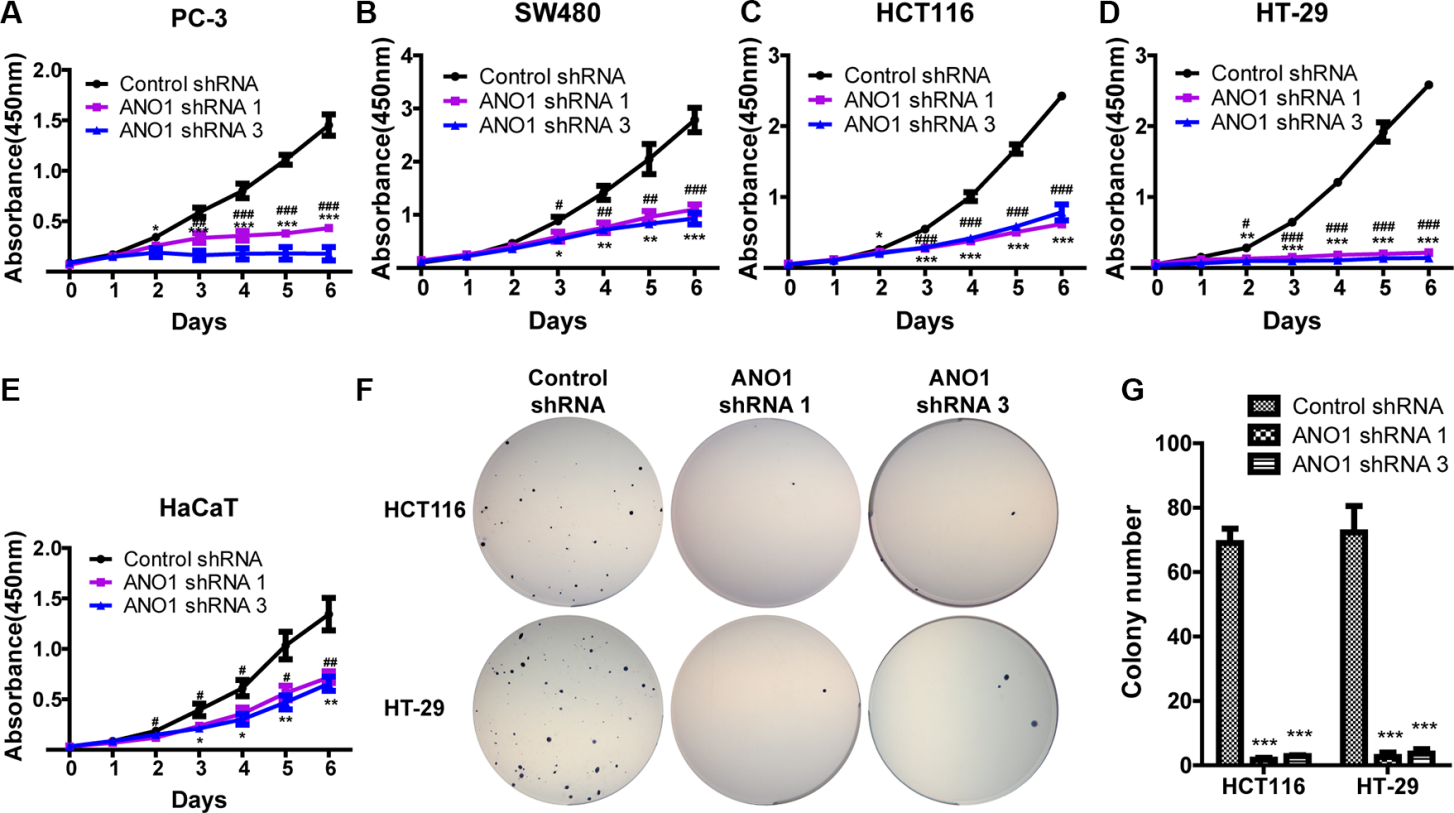
Silencing ANO1 inhibits cell proliferation (**A**–**E**) Cell proliferation of the indicated cell lines was assessed by CCK8 assay. Three days after lentiviral infection of ANO1 shRNAs, cells were re-plated into 96-well plates and cell proliferation was measured every day for six days. Silencing ANO1 resulted in significant inhibition of cell viability in a time-dependent manner. Data are expressed as mean ± SEM; *n* = 4; ^#^*P* < 0.05, ^##^*P* < 0.01 and ^###^*P* < 0.001 are for statistical comparisons of ANO1 shRNA 1 versus control shRNA; ^*^P < 0.05, ^**^ *P* < 0.01 and ^***^ *P* < 0.001 are for statistical comparisons of ANO1 shRNA 3 versus control shRNA. (**F**) Representative images of colony formation in soft agar assay. Knockdown of ANO1 by ANO1 shRNA1 or shRNA3 decreased the colony-formation capacity of HCT116 and HT-29 as compared with control shRNA. (**G**) Quantitative analysis of colony numbers from F. Data are expressed as mean ± SEM; *n* = 3; ^***^P < 0.001.

Having screened the expression of ANO1 in the selected cell lines, we intended to evaluate its biological roles by gene silencing. Since lentivirus can transduce a wide range of cell types with high efficiency and integrate into the host genome, resulting in long-term expression of the transgene, we constructed lentiviral vectors expressing ANO1 shRNAs or control shRNA according to the sequences previously used in our lab. Lentiviral particles were used to silence the expression of ANO1 in these cell lines. Three days after infection, cells were re-plated into 96-well plates and cell proliferation was measured by CCK8 every day for six days. As shown in Figure 2A–2E, knockdown of ANO1 resulted in significant inhibition of cell proliferation in a time-dependent manner compared with control shRNA. Similar results were obtained in all cell lines tested, whether tumor cell lines or normal HaCaT cells.

To further confirm the effect of ANO1 on proliferation, we selected two colon cancer cell lines, HCT116 and HT-29, and observed the colony formation in soft agar after ANO1 silencing. Fewer colonies were found in groups expressing ANO1 shRNAs (Figure 2F). Quantitative analysis of colony number revealed that knockdown of ANO1 significantly reduced the colony formation ability of both HCT116 and HT-29 cells (Figure 2G).

### Silencing ANO1 causes retardation of the cell cycle procession

**Figure 3:**
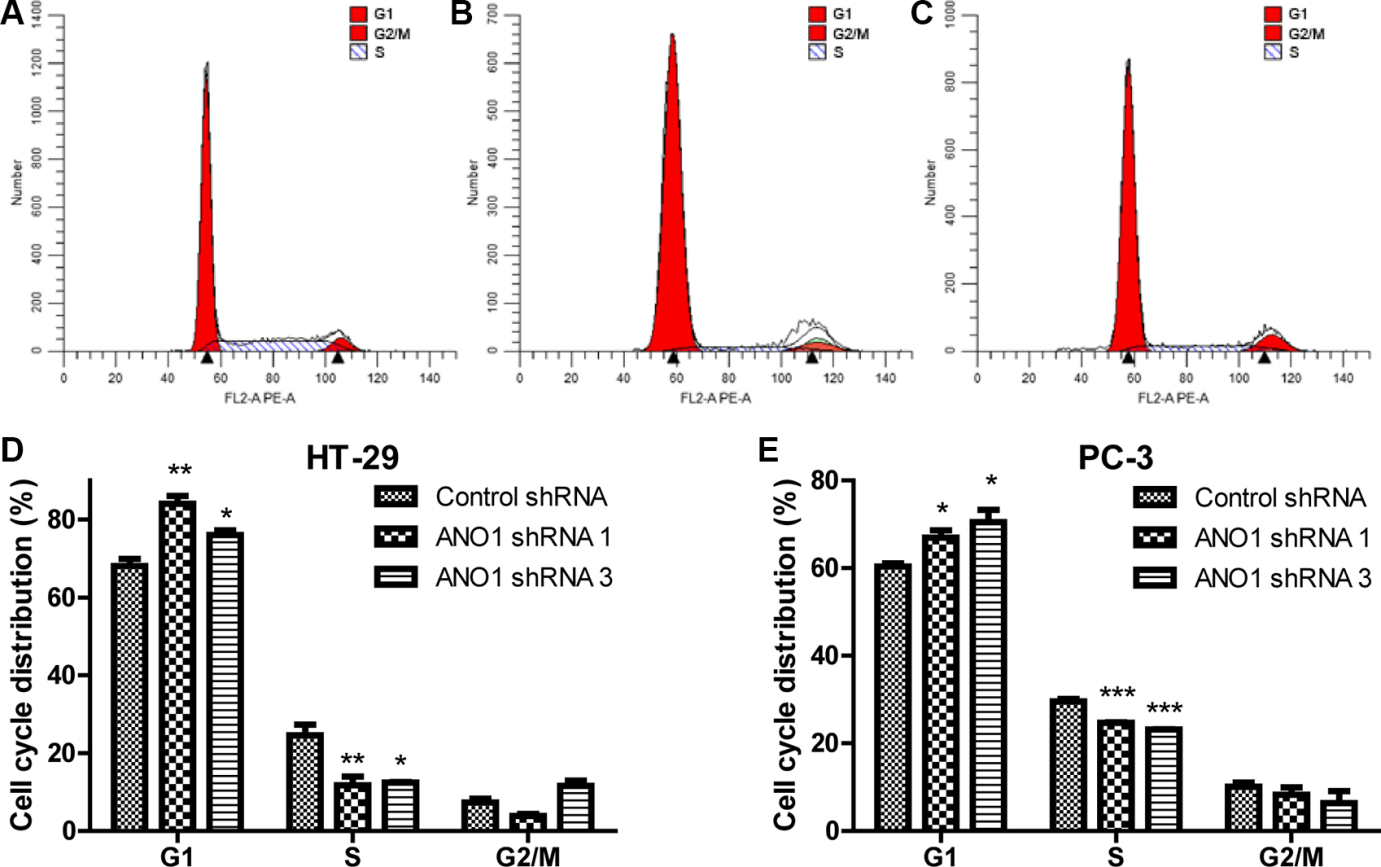
Flow cytometric analysis of the cell cycle distribution in colon cancer HT-29 and prostate cancer PC-3 cells Flow cytometric analysis of the cell cycle distribution in colon cancer HT-29 cells infected with control shRNA virus (**A**), ANO1 shRNA 1 virus (**B**) and ANO1 shRNA 3 virus (**C**) respectively. Bar graphs showing an increase of G1 phase and a decrease of S phase in cell cycle for the percentage of indicated cells after ANO1 silencing in HT-29 (**D**) and PC-3 (**E**) cell lines. Data are expressed as mean ± SEM; *n* = 3; ^*^P < 0.05; ^**^P < 0.01; ^***^P < 0.001.

The decrease of cell proliferation can result from either slow proliferation rate or increased cell death. To confirm which mechanism underlying the decrease of cell proliferation, we measured the cell cycle distribution using flow cytometry analysis. In colon cancer HT-29 cells, silencing ANO1 resulted in significant increase of the cell number at G1 phase and a decrease of the cell number at S phase. The percentage of cells in G1 phase increased from 68.08% to 84.07% and 76.02% by ANO1 shRNA 1 and shRNA 3, respectively, while the percentage of cells in S phase decreased from 24.6% to 11.72% and 12.42%, respectively (Figure 3A–3D). Similar results were obtained in PC-3 cells treated with ANO1 shRNAs (Figure 3E). These results indicated that knocking-down ANO1 arrested the cells at G1 phase of the cell cycle, thus caused the inhibitory effects on cell growth.

**Figure 4:**
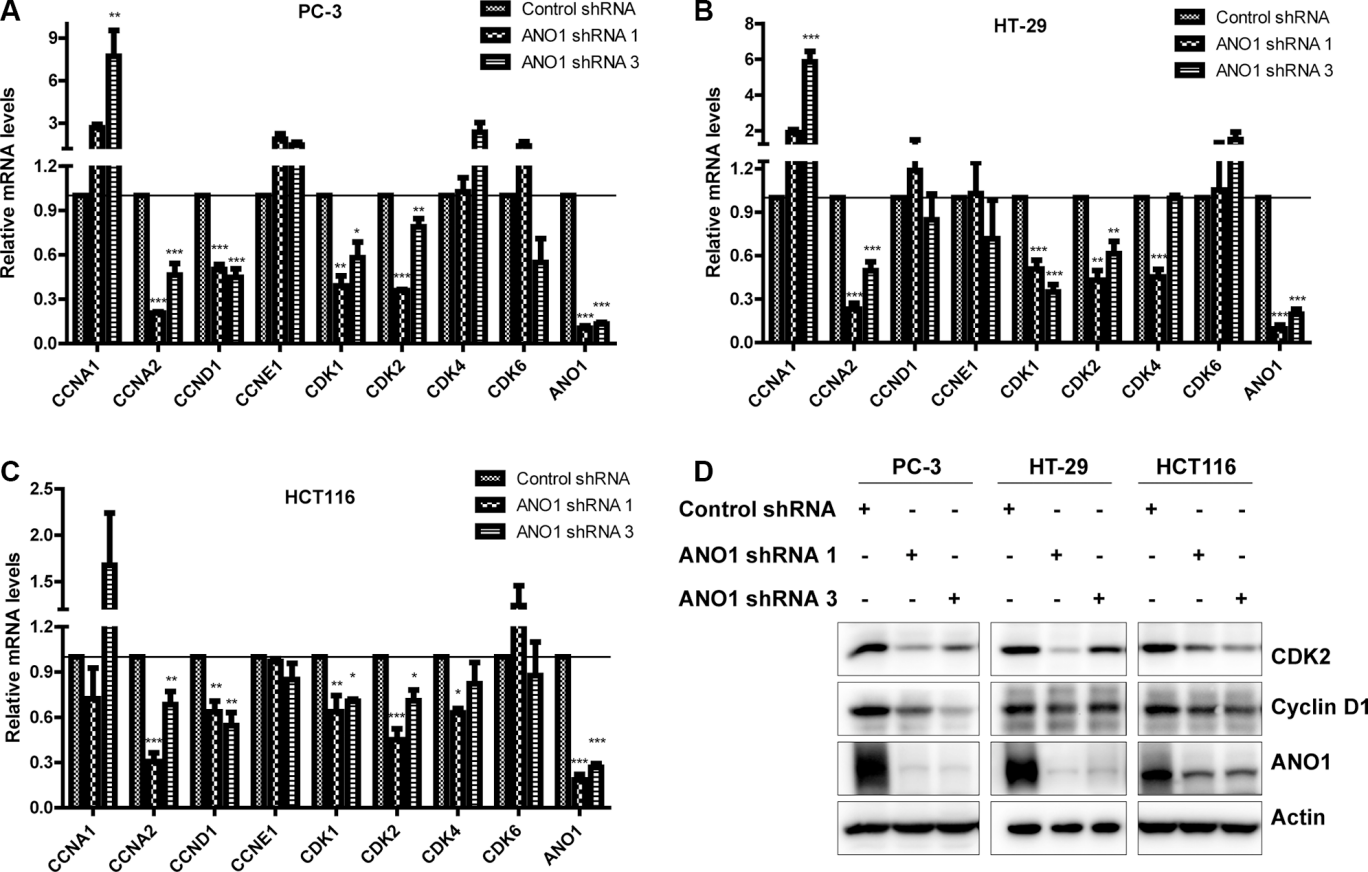
Analysis of mRNA and protein levels of cyclins and CDKs in PC-3, HT-29 and HCT116 cells after ANO1 knockdown Four days after lentiviral infection of ANO1 shRNAs, the mRNA levels of several cyclins and CDKs were examined by real-time PCR. Bar graphs showing reduced expressions of cyclin D1 (CCND1), cyclin A2 (CCNA2), CDK1 and CDK2 in prostate cancer PC-3 cells (**A**), decreased expressions of cyclin A2, CDK1 and CDK2 in colon cancer HT-29 cells (**B**), and reduced expressions of cyclin D1, cyclin A2, CDK1 and CDK2 in colon cancer HCT116 cells (**C**). Data are expressed as mean ± SEM; *n* = 3–4; ^*^P < 0.05; ^**^P < 0.01; ^***^P < 0.001. (**D**) Western blot shows a decreased expression of CDK2 and cyclin D1 in the indicated cells after knockdown of ANO1.

Cyclins and CDKs are key regulators of the cell cycle, we therefore detected the changes of these molecules at mRNA level by real-time PCR. In prostate cancer PC-3 cells, silencing ANO1 reduced the expression of cyclin D1 (CCND1), cyclin A2 (CCNA2), CDK1 and CDK2 (Figure 4A). As shown in Figure 4B, the expression of cyclin A2, CDK1 and CDK2 in colon cancer HT-29 cells was decreased upon the knockdown of ANO1 (Figure 4B). Similar reduction of cyclins and CDKs was found in colon cancer HCT116 cells (Figure 4C). We also detected the change of these molecules at protein level, and found that silencing ANO1 decreased protein expression of CDK2 and cyclin D1 in PC-3, HT-29 and HCT116 cells (Figure 4D).

### Silencing ANO1 induces cell apoptosis in prostate cancer and colon cancer cells

**Figure 5:**
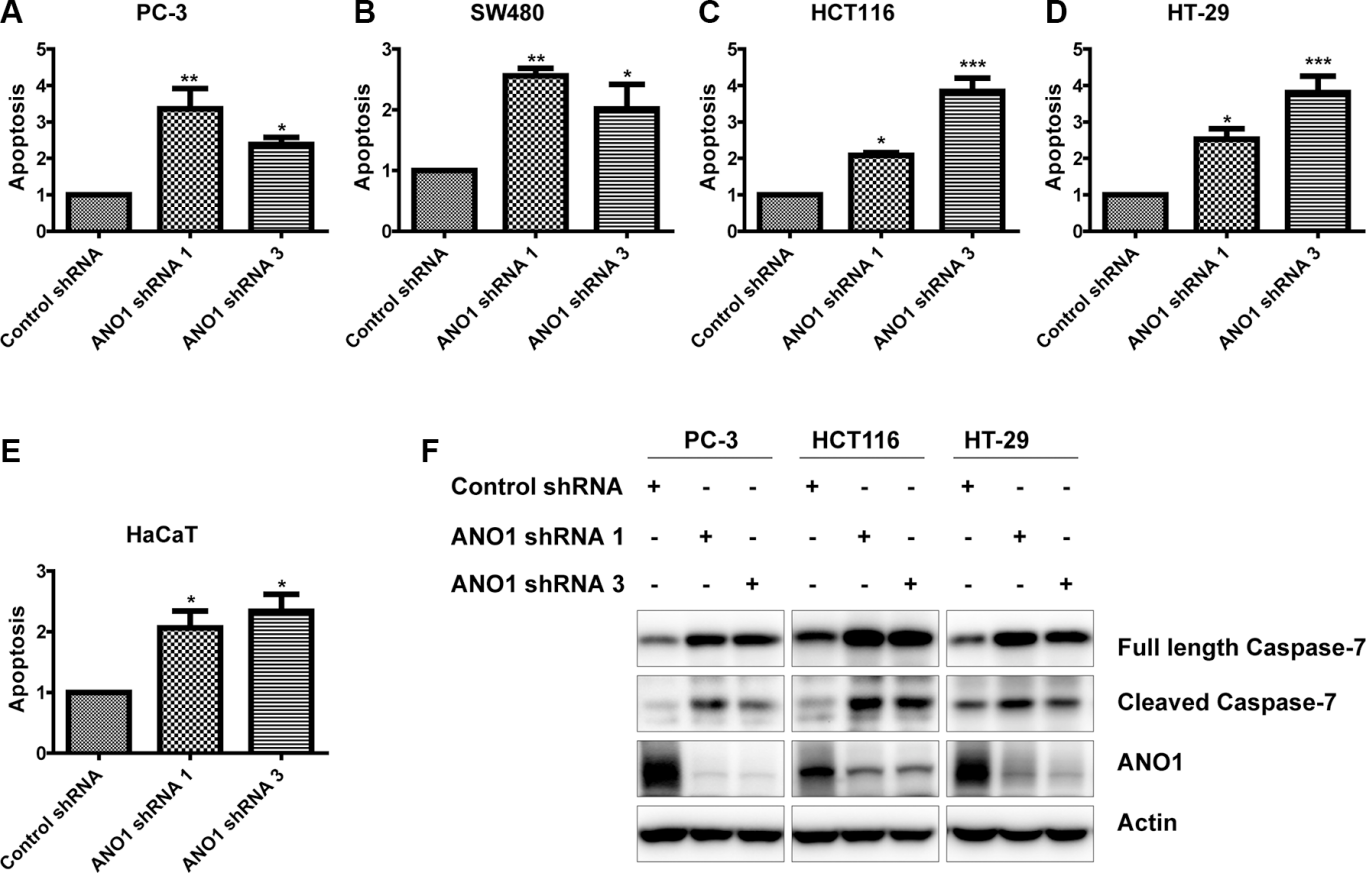
Knockdown of ANO1 induces cell apoptosis in prostate cancer and colon cancer cells Four days post-infection, cell apoptosis was determined by ELISA in prostate cancer PC-3 cells (**A**) and colon cancer cells SW480 (**B**), HTC116 (**C**) and HT-29 (**D**) as well as skin HaCaT cells (**E**). Data are expressed as mean ± SEM; *n* = 3–4; ^*^P < 0.05; ^**^P < 0.01; ^***^P < 0.001. (**F**) Western blot showed increased Caspase-7 expression in the indicated cells after knockdown of ANO1.

To investigate the effects of ANO1 on cell apoptosis of cancer cell lines, we carried out an ELISA assay to determine the amount of nucleosomes in the cytoplasmic fraction of cell lysates on the fourth day after lentiviral infection of ANO1 shRNAs. As shown in Figure 5A–5D, knockdown of ANO1 induced apoptosis in prostate cancer PC-3 cells and colon cancer cell lines SW480, HCT116 and HT-29, as compared with cells expressing control shRNA. In Figure 5E, silencing ANO1 in skin HaCaT cells also resulted in an increase of apoptosis, suggesting the critical role of ANO1 in skin physiology. Caspases, a family of cysteine acid proteases, are central regulators of apoptosis. We detected the changes of some effector caspases by Western blot. We found an increased expression of full-length and cleaved caspase-7 upon knockdown of ANO1 in PC-3, HCT116 and HT-29 cells (Figure 5F). These results show that ANO1 functions as a protein that promotes cell survival.

### ANO1 inhibitors decrease cell viability and suppress cell migration

**Figure 6:**
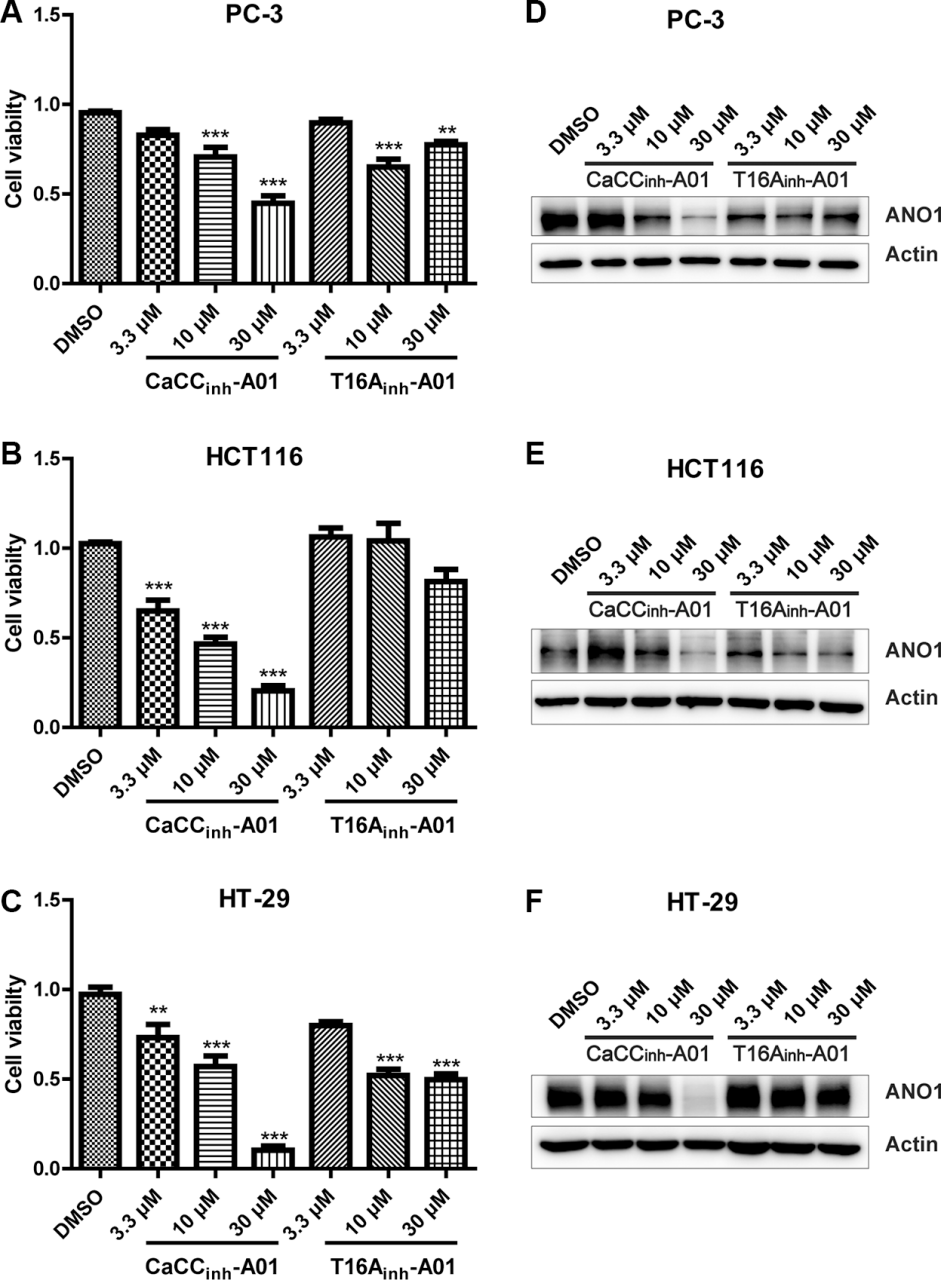
Effects of pharmacological inhibition of ANO1 on cell viability of PC-3, HCT116 and HT-29 Cells were treated with different concentrations of ANO1 blockers T16A_inh_-A01 or CaCC_inh_-A01 for 72 h. Cell viability was measured by CCK8 assay. Inhibition of ANO1 with CaCC_inh_-A01 decreased the viability of PC-3 (**A**), HCT116 (**B**) and HT-29 (**C**) cells in a dose-dependent manner, whereas T16A_inh_-A01 showed a weak inhibitory effect on cell viability compared with CaCC_inh_-A01. Data were normalized to the DMSO-treated samples. Data are expressed as mean ± SEM; *n* = 4; ^**^P < 0.01; ^***^P < 0.001. Effects of CaCC_inh_-A01 and T16A_inh_-A01 on ANO1 protein levels were examined by Western blot after treatment of different concentrations of inhibitors or DMSO for 72 h, and representative Western blots are shown (**D**–**F**).

Having shown that genetic ablation of ANO1 inhibited cell proliferation and induced cell apoptosis, we wondered whether ANO1 inhibitors could show any similar effects. Two ANO1 inhibitors, T16A_inh_-A01 and CaCC_inh_-A01 were used to test whether the chloride channel activity of ANO1 is required for cell proliferation. Cells were treated with various concentrations of inhibitors for 72 h before cell viability was measured by CCK8 assay. The results demonstrated that T16A_inh_-A01 had a little inhibitory effect on cell viability at a high concentration of 30 μM, while CaCC_inh_-A01 could reduce the viability of PC-3, HCT116 and HT-29 cells in a dose-dependent manner (Figure 6A–6C). The effects of CaCC_inh_-A01 and T16A_inh_-A01 on ANO1 protein levels were examined by Western blot after treating different concentrations of ANO1 inhibitors for 72 h. CaCC_inh_-A01 markedly decreased ANO1 protein levels in concentration-dependent manner, whereas inhibitor T16A_inh_-A01 had a weak effect (Figure 6D–6F).

**Figure 7:**
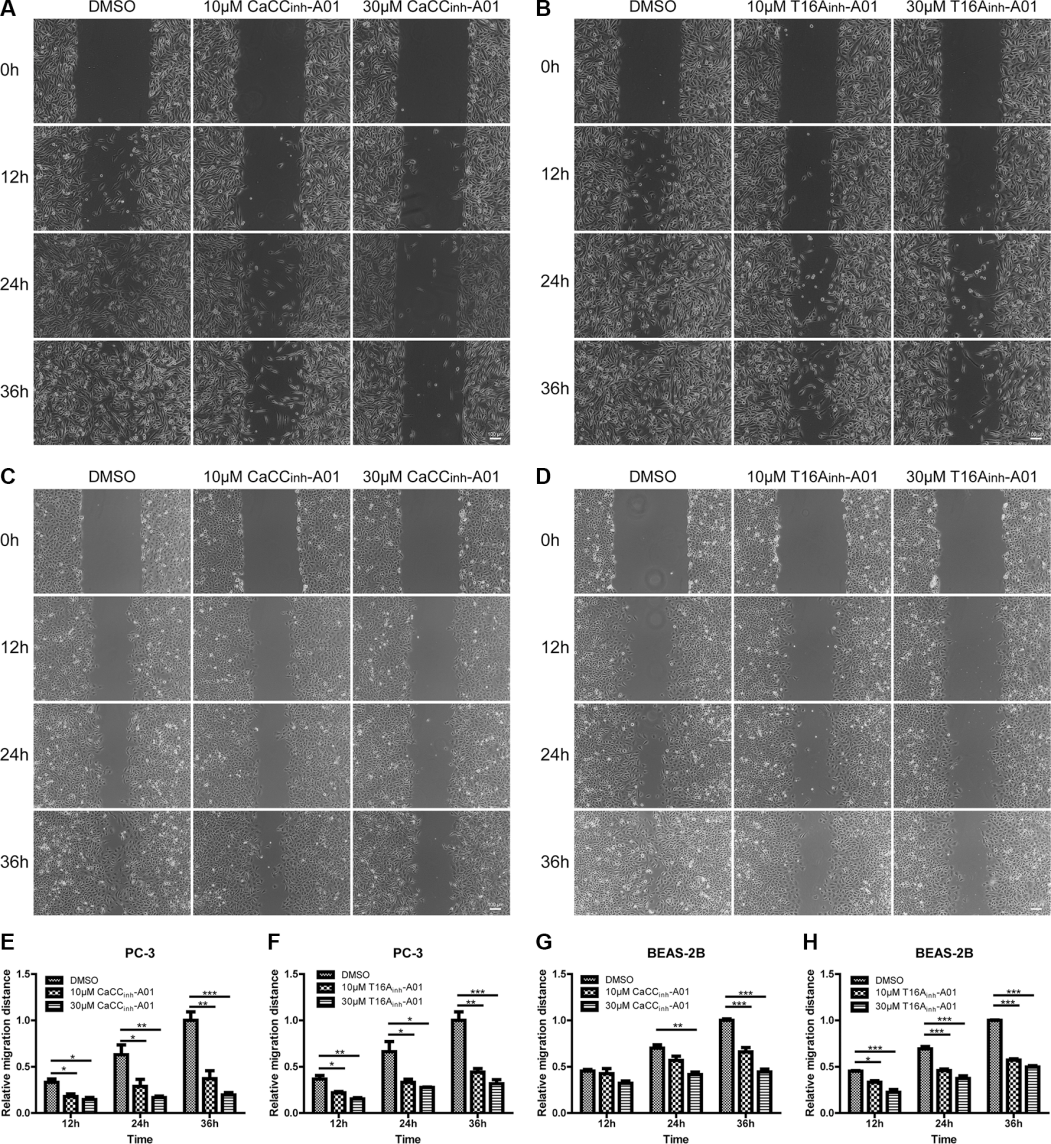
Suppression of cell migration of PC-3 and BEAS-2B cells by ANO1 inhibition in wound-healing assay Cell migration was assessed by wound-healing assay in the presence of ANO1 inhibitor or DMSO control. Images of wound closure in prostate cancer PC-3 cells treated with different concentrations of CaCC_inh_-A01 (**A**) or T16A_inh_-A01 (**B**) were taken at 0 h, 12 h, 24 h and 36 h. Representative images of bronchial epithelial BEAS-2B cells treated with CaCC_inh_-A01 (**C**) or T16A_inh_-A01 (**D**) were presented. Scale bar: 100 μm. (**E**–**H**) Bar graphs of panel A–D showing treatment of PC-3 and BEAS-2B cells with CaCC_inh_-A01 or T16A_inh_-A01 suppressed cell migration. Data are expressed as mean ± SEM; *n* = 3; ^*^ *P* < 0.05; ^**^ *P* < 0.01;^***^ *P* < 0.001.

In addition, we performed wound-healing assay to investigate the effects of ANO1 inhibitors on cell migration. Treatment of PC-3 cells with CaCC_inh_-A01 or T16A_inh_-A01 inhibited the wound closure as compared to DMSO group (Figure 7A and 7B). Similar results were obtained in BEAS-2B cells (Figure 7C and 7D). Quantitative analysis of the relative migration distance showed that in PC-3 cells, 30 μM CaCC_inh_-A01 and 30 μM T16A_inh_-A01 reduced cell migration by 80.5% and 68.4% at 36 h, while in BEAS-2B cells, the inhibitory rate was 55.6% and 50.3% respectively (Figure 7E–7H).

## DISCUSSION

The purpose of this study was to investigate the role of calcium-activated chloride channel ANO1 in epithelium-originated cancer cells that highly express the channel. Previous investigations from others and ours have shown that ANO1 is amplified or overexpressed in various cancers including head and neck squamous cell carcinoma (HNSCC), prostate cancer, breast cancer, colon cancer and lung adenocarcinoma, making it a promising tumor marker [18, 19, 23]. However, there are also reports that ANO1 affects neither cell proliferation nor migration, and even inhibits cell migration [23, 24]. The inconsistent results suggest that ANO1 may exert biological effects that are cell type-dependent. In this study, we find that knockdown of ANO1 inhibits cell proliferation and induces cell apoptosis of four different epithelial tumor cell lines that highly express ANO1 channels. Consistent with our findings, ANO1 has been reported to be a regulator of proliferation in interstitial cells of Cajal (ICC), pacemaker cells of the gut. ANO1 is expressed abundantly and specifically in ICC and plays fundamental role in the generation of slow waves in gastrointestinal smooth muscles [7]. Mice lacking ANO1 had significantly fewer proliferating ICC in the small intestine, suggesting that ANO1 regulates proliferation of ICC [25]. Recently, Cha et al. found that ANO1 is essential for testosterone-induced prostate hyperplasia, and selective knockdown of ANO1 inhibits DHT-induced cell proliferation *in vitro* and reduces prostate enlargement *in vivo* [26]. This finding is consistent with our observations that ANO1 promotes proliferation of prostate cancer cells.

Although we have not extensively investigated the underling mechanism of ANO1 in proliferation, it is likely that some common signaling pathways are shared partially in these epithelium-originated cells in which ANO1 is highly expressed and promotes proliferation. Evidence from breast cancer shows that ANO1 is involved in oncogenic signaling by activating EGFR and CAMK pathways to promote cancer progression [15]. In the HNSCC cell line UM-SCC1 and colon cancer SW620 cells, ANO1 promotes cell proliferation by activating MAPK signaling for progression of cell cycle [18, 21]. Since cyclins and CDKs are key regulators of the cell cycle [27], we have also detected the changes of these molecules and the cell cycle distribution. Our results show decreased expression of some cyclins and CDKs and the cell cycle arrest at G1 phase after ANO1 knockdown, indicating that cell cycle arrest may contribute partially to the decrease of proliferation. Our observation also shows that ANO1 silencing induces apparent apoptosis, which is likely another reason responsible for decreased proliferation.

There is evidence that the chloride channel activity of ANO1 is required for its effects on cell viability. Treatment with channel inhibitors or mutations in critical residues for anion permeability abrogate cell proliferation in many cell lines including breast cancer and HNSCC [23]. However, contrary reports also show that inhibition of ANO1 function alone is not sufficient to diminish proliferation of ANO1-dependent cancer cells. The ANO1 inhibitor CaCC_inh_-A01 decreases cancer cell proliferation by facilitating degradation of ANO1 protein, while T16A_inh_-A01, another small molecule inhibitor of ANO1, cannot inhibit the proliferation of these cell lines, although both compounds have been reported to inhibit ANO1 activity [28–31]. Structure-activity analysis reveals that both the carboxylic acid and hydrophobic bulk present in CaCC_inh_-A01 are necessary for its effect on cell proliferation [30]. Consistent with the latter view, we found that treatment with CaCC_inh_-A01 reduces cell viability in a dose-dependent manner whereas the inhibitor T16A_inh_-A01 shows a little inhibitory effect on cell proliferation. Furthermore, a decrease in ANO1 protein level was also observed. It is reasonable to assume that in these cell lines CaCC_inh_-A01 also reduces ANO1 protein levels by facilitating endoplasmic reticulum-associated, proteasomal degradation of ANO1, thus inhibiting cell proliferation.

The different effects of the two ANO1 inhibitors on cell proliferation and migration suggest that distinct mechanism may be involved in the two processes. As discussed above, inhibition of the channel activity is not sufficient to affect cell proliferation, and ANO1 may exert its pro-survival properties via interaction with other proteins. Indeed ANO1 has been shown to associate with EGFR to facilitate the EGFR-signaling and regulate HNSCC cell proliferation [32]. The protein degradation of ANO1 induced by CaCC_inh_-A01 may impair this interaction, thus diminishing its pro-survival effects. The process of cell migration comprises cell swelling at the leading edge and subsequent cell shrinkage at the rear part of the cell. The increase in intracellular calcium will activate calcium-activated chloride channels and calcium-activated potassium channels at cell rear edge to release intracellular anion ions and potassium ions, resulting in shrinkage of cell volume [33, 34]. Because ANO1 is activated by cell swelling and involved in regulatory volume decrease (RVD) [35, 36], we propose that ANO1 may affect migration by regulating cell shape or volume, and inhibiting the channel activity impairs migration ability.

In summary, we have shown in this study that knockdown of ANO1 inhibits cell proliferation and induces cell apoptosis in different epithelium originated cancer cells that highly express ANO1 channels. Pharmacological inhibition of ANO1 with inhibitor CaCC_inh_-A01 reduces cell viability and suppresses cell migration. Our findings indicate that ANO1 promotes cell proliferation and migration, and the pro-survival properties of ANO1 are shared by different types of epithelial cancers, suggesting the critical role of calcium-activated chloride channel ANO1 in the pathophysiology of epithelial cells.

## MATERIALS AND METHODS

### Cell culture

Human prostate cancer cell line PC-3, human bronchial epithelial cell line BEAS-2B and colon cancer cell lines SW480, HCT116 and HT-29 were cultured in RPMI-1640 medium (Invitrogen) supplemented with 10% FBS (Gibco). Human keratinocyte HaCaT cells and human embryonic kidney 293LTV cells were maintained in DMEM (Invitrogen) supplemented with 10% FBS. Normal breast epithelium MCF 10A cells were maintained in DMEM/F-12 medium (Invitrogen) supplemented with 5% horse serum (Gibco), 20 ng/ml human recombinant EGF (Peprotech), 0.5 μg/ml hydrocortisone (M&C Gene Technology), 100 ng/ml cholera toxin (M&C Gene Technology), 10 μg/ml insulin (Sigma). The cells were cultured at 37°C with 5% CO_2_ in a humidified incubator. PC-3, 293LTV, MCF 10A cells, SW480, HCT116 and HT-29 cells were gifts from Dr. Hongquan Zhang at the Department of Anatomy, Histology and Embryology, Peking University Health Science Center. BEAS-2B cells were gifts from Dr. Bo Zhang at the Department of Pathology, Peking University Health Science Center. The HaCaT cell line was originally obtained from the American Type Culture Collection.

### Constructions of lentiviral plasmids and preparations of lentivirus

The lentiviral vector pLKO.1 was used to construct ANO1 shRNAs and control shRNA plasmids. The shRNA oligos were designed according to the target sequences previously used in our lab [16]. The oligo sequences of ANO1 shRNAs and control shRNA were as follows: ANO1 shRNA 1 forward, CCGGGACGT GTACAAAGGCCAAGTACTCGAGTACTTGGCCTTTG TACACGTCTTTTTG; ANO1 shRNA 1 reverse, AATTC AAAAAGACTGTACAAAGGCCAAGTACTCGAGTAC TTGGCCTTTGTACACGTC; ANO1 shRNA 3 forward, CCGGGACGAAGAAGATGTACCACATCTCGAGATG TGGTACATCTTCTTCGTCTTTTTG; ANO1 shRNA 3 reverse, AATTCAAAAAGACGAAGAAGATGTACCA CATCTCGAGATGTGGTACATCTTCTTCGTC; control shRNA forward, CCGGGACGAGTGGTCTAGTTG AGAACTCGAGTTCTCAACTAGACCACTCGTCTTTT TG; control shRNA reverse, AATTCAAAAAGACGAGT GGTCTAGTTGAGAACTCGAGTTCTCAACTAGACC ACTCGTC. The oligos were annealed and cloned into the Age*I* and EcoR*I* sites of pLKO.1 vector. For lentiviral production, 293LTV cells were cotransfected using Lipofectamine 2000 (Invitrogen) with pLKO.1 shRNA and packaging plasmids. The culture medium was replaced after 16 h, and supernatants were collected 48 h and 72 h after transfection. Target cells were infected with lentivirus in the presence of 8 μg/ml Polybrene (M&C Gene Technology).

### RNA extraction and real-time PCR

Total RNA was isolated from cells by Trizol reagent (Invitrogen). Two micrograms of RNA was reverse transcribed to cDNA using the GoScript™ Reverse Transcription System (Promega). Real-time PCR was set up using SYBR Green mix (Promega) with the following PCR conditions: 95°C for 2 minutes; 95°C for 15 seconds, and 60°C for 1 minute, for 40 cycles. The primer sequences were as follows: ANO1 forward, GAGCCAAAGACATCGGAATCTG; ANO1 reverse, TGA AGGAGATCACGAAGGCAT; CCNA1 forward, ACATG GATGAACTAGAGCAGGG; CCNA1 reverse, GAGTGT GCCGGTGTCTACTT; CCNA2 forward, CGCTGGCG GTACTGAAGTC; CCNA2 reverse, GAGGAACGGTG ACATGCTCAT; CCND1 forward, AGAGGCGGAGGA GAACAAAC; CCND1 reverse, GGCACAGAGGGCAAC GAAG; CCNE1 forward, TCGGCCTTGTATCATTTC TCGTC; CCNE1 reverse, GCTCCCCGTCTCCCTTATAA CC; CDK1 forward, GGATGTGCTTATGCAGGATTCC; CDK1 reverse, CATGTACTGACCAGGAGGGATAG; CDK2 forward, GTGGGCCCGGCAAGATTTTAG; CDK2 reverse, GCCGAAATCCGCTTGTTAGGG; CDK4 forward, CTGGTGTTTGAGCATGTAGACC; CDK4 reverse, GATCCTTGATCGTTTCGGCTG; CDK6 forward, CCAGATGGCTCTAACCTCAGT; CDK6 reverse, AACTTCCACGAAAAAGAGGCTT; Actin forward, AGAAGGATTCCTATGTGGGCG; Actin reverse, GGATAGCACAGCCTGGATAGCA. Expression of various genes was determined by the comparative Ct method (2^−ΔΔCt^). All genes were normalized to Actin levels.

### Western blot analysis

Cell lysates were prepared using Triton X-100 lysis buffer (150 mM NaCl, 20 mM Tris, 1% Triton X-100, 1% sodium deoxycholate, 0.1% SDS, 10 mM EDTA) containing cocktail protease inhibitor (Roche Applied Science). Protein samples were denatured at 95°C for 5 minutes, separated by SDS-PAGE and then transferred to PVDF membranes (Millipore). After blocking with 5% milk for 1 hour, the membranes were incubated with primary antibodies against ANO1 (1:1000; ab53212, Abcam), Caspase-7 (1:1000; 12742, Cell Signaling Technology), CDK2 (1:500; sc-6248, Santa Cruz), Cyclin D1 (1:1000; 2978, Cell Signaling Technology) or actin (1:2000; TA-09, Zhongshan golden bridge) overnight at 4°C. After extensive washing in TBS buffer, the membranes were incubated with horseradish peroxidase-conjugated secondary antibody for 1 hour at room temperature. The signal was detected using an ECL Western blotting detection system (Millipore).

### Cell proliferation assay

Cells were re-plated into 96-well plates at a density of 2000 cells per well 3 days after ANO1 shRNA infection, and cell proliferation was measured after cell adherence and on the following six days by Cell Counting Kit-8 (Dojindo Laboratories, Japan). Briefly, 10 μl of the Cell Counting Kit solution was added into each well and the cells were further incubated for 2 hours at 37°C. Then the absorbance was measured at 450 nm with a microplate reader (Thermo Scientific). For ANO1 inhibitors, cell viability was determined 72 hours after treatment with different concentrations of T16A_inh_–A01 (Sigma) or CaCC_inh_–A01 (Sigma).

### Soft agar colony formation assay

Colony formation assay in soft agar was carried out in 6-well plates. The base layer was made by mixing 1.2% LMP agarose (Invitrogen) and equivalent volume of 2× medium supplemented with 20% FBS. Then cells expressing ANO1 shRNA or control shRNA were harvested and suspended in medium containing 0.4% agarose and plated over the base layer in triplicate at a density of 3000 cells per well. Three weeks later, cells were stained with NBT (Sigma) overnight in the incubator when colonies were visible. Images were taken after staining and the number of blue colonies was counted.

### Cell-cycle analysis

Cells were detached using trypsin-EDTA, washed with PBS and fixed overnight in 70% ethanol at −20°C. The fixed cells were washed twice with PBS, treated with 10 μg/ml RNase A for 30 minutes at 37°C before stained with 50 μg/ml PI. The stained cells were analyzed by flow cytometry (Beckman).

### Apoptosis detection by ELISA

Three days after lentiviral infection, cells were re-plated into 12-well plates at the density of 7 × 10^4^ per well, and further incubated for one day. Then cell apoptosis was detected by Cell Death Detection ELISA kit (Roche) according to the manufacturer's instructions. Cells were lysed with 0.5 ml of lysis buffer at room temperature for 30 minutes. Then cell lysates were centrifuged at 200 × g for 10 minutes at 4°C. Transfer 20 μl of the supernatant into the streptavidin coated MP, add 80 μl of the immunoreagent containing Anti-DNA-POD and Anti-histone-biotin to each well and incubate at room temperature for 2 hours with shaking. Wash the wells for three times, then add 100 μl ABTS and incubate for 10–20 minutes before the absorbance was measured at 405 nm.

### Wound-healing assay

Cell migration was measured by *in vitro* wound-healing assay. Cells were seeded in 6-well plates until confluent and starved for 12 hours in serum free medium. Cells were scraped with a sterile 200 μl tip and washed twice with PBS. The cells were photographed at 0 h, 12 h, 24 h and 36 h under an inverted microscope (Olympus). The relative migration distance was determined by a ratio of average migration distance in cells treated with ANO1 inhibitors versus that in DMSO group.

### Statistical analysis

All experiments were repeated for at least three times, and data were presented as mean ± SEM. Statistical analyses were performed in GraphPad Prism 5 using one-way ANOVA. *P* value of less than 0.05 was considered statistically significant.
